# 

**Published:** 1882-09

**Authors:** 


### CONTENTS:

ART. I.—The American Dental Association.......................................193
ART. II—The Lancet in Dentition. By Thos. S. Sozinskey, M. D., Ph., D.........203
ART. III.—Erosion: The Probable Causes and Treatment. By J. J. Bailey L. D. S. 211
ART. IV.—Porcelain Crowns. By Dr. W. H. Shulze.................................219
ART. V.—Combination of Tin and Gold Fillings By A H. Thompson D. D. S....224
ART. VI.—Effects of Irritation on Motor Region of the Brain......................229
ART. VII.—Color Relation of Metals.......................................232
Dental Department of the University of Maryland.............................233

### EDITORIAL, ETC.

Pulp Treatment.............................................235
Improvements in Vulcanite...................................235

### MONTHLY SUMMARY.

American Dyspeysia..............................................235
Rest in Treatment of Heart Disease................................236
Food Adulteration.............................................237
Tobacco Smoking—An Experiment.................................238
A Marvel of Surgery...........................................239
Nitrous Oxide..................................................239
Oxalate of Lime Calculus.......................................239
Backache: Its Causes..........................................240

